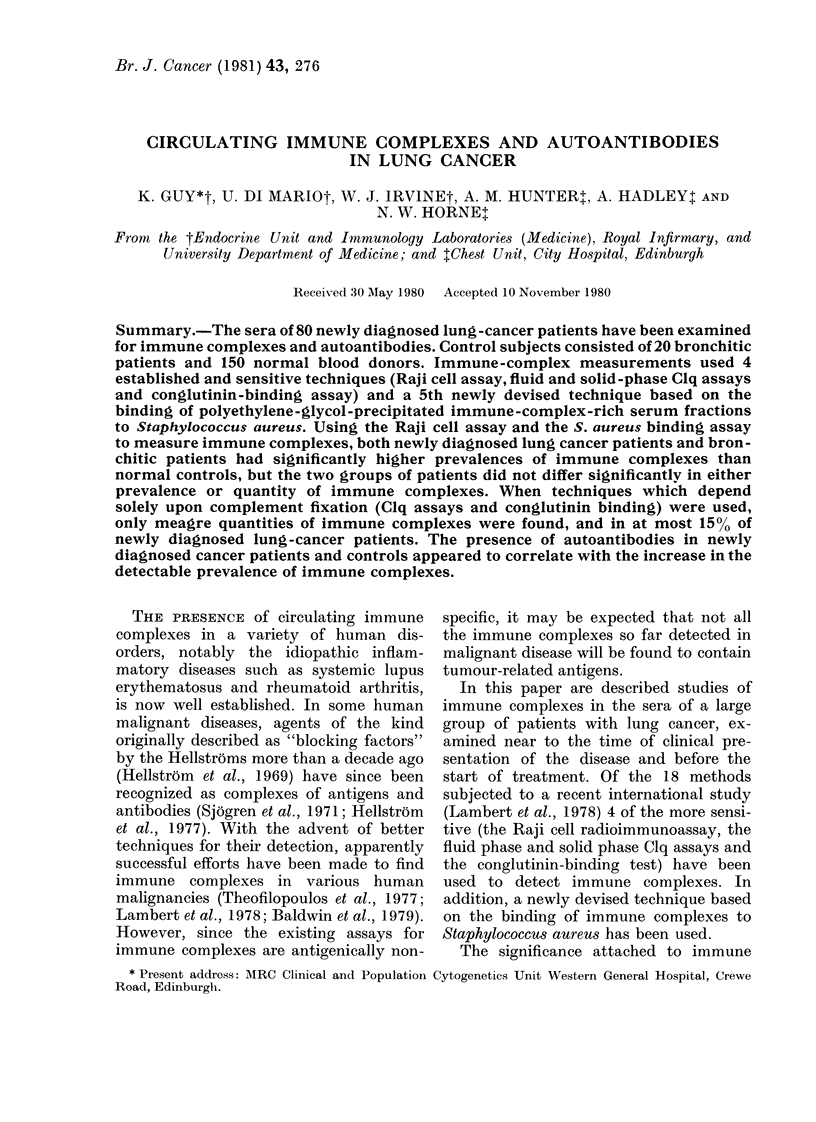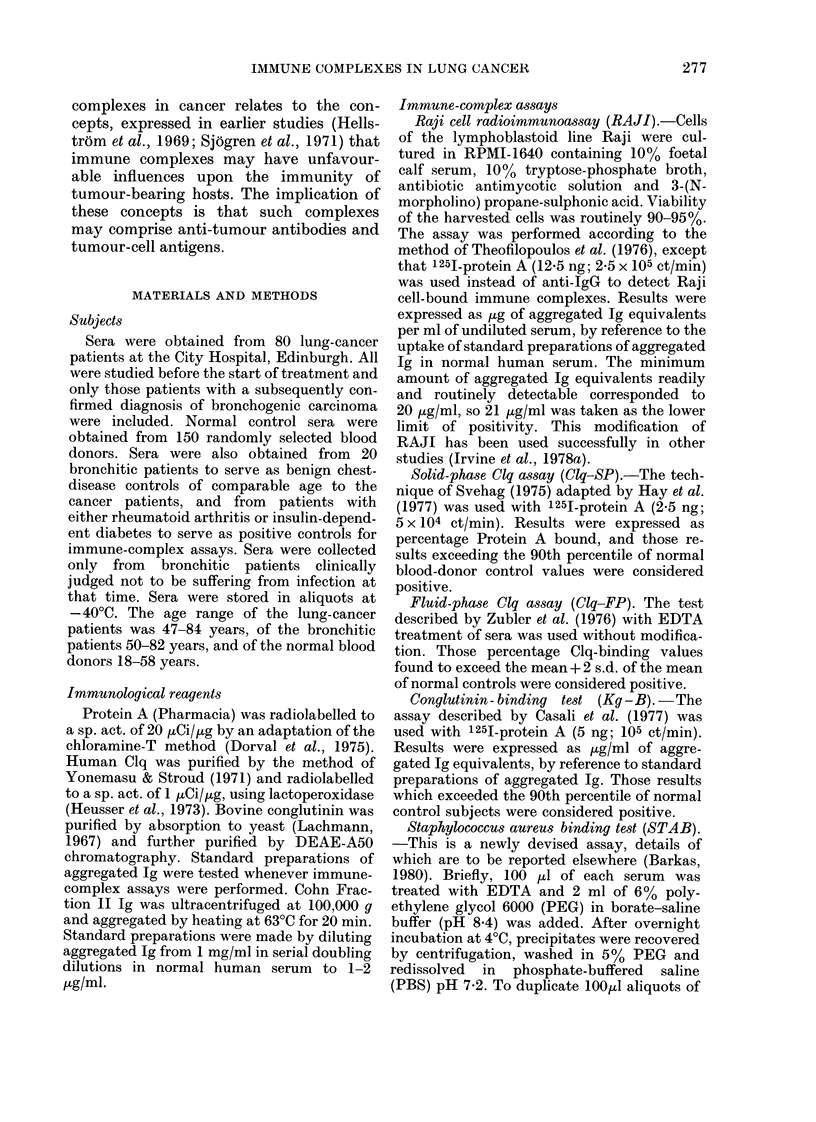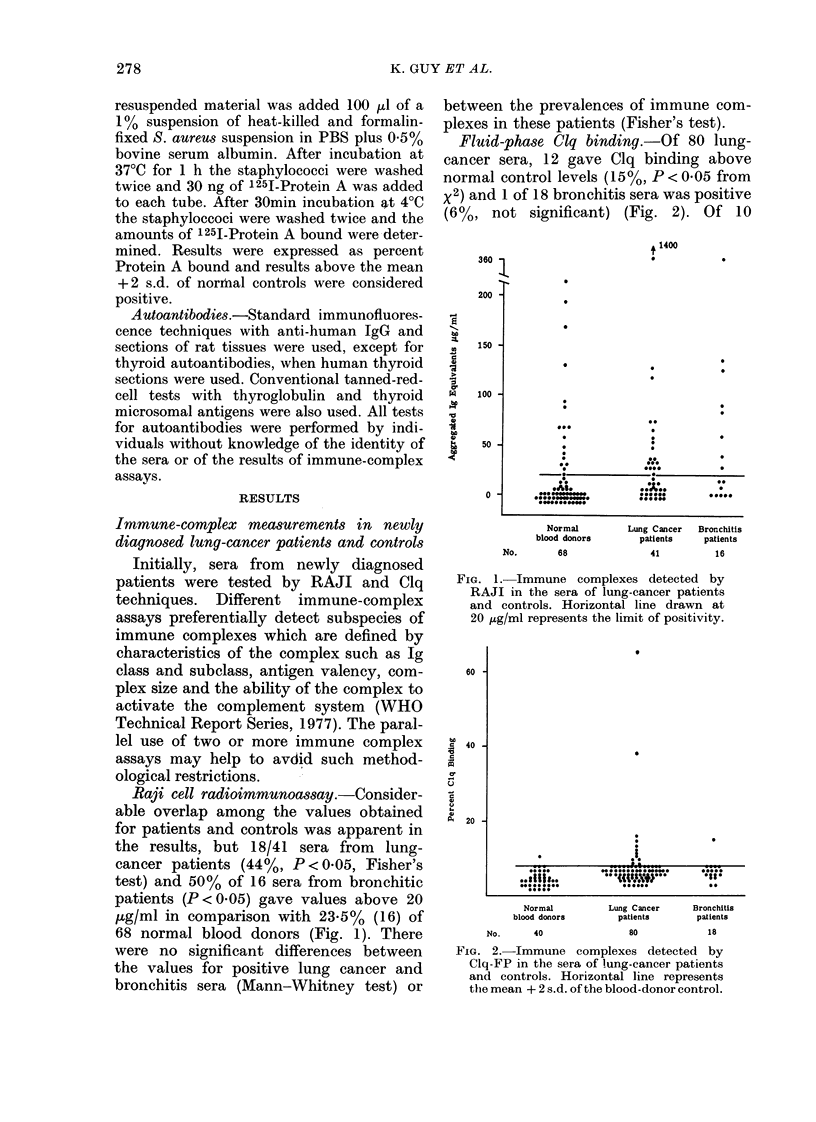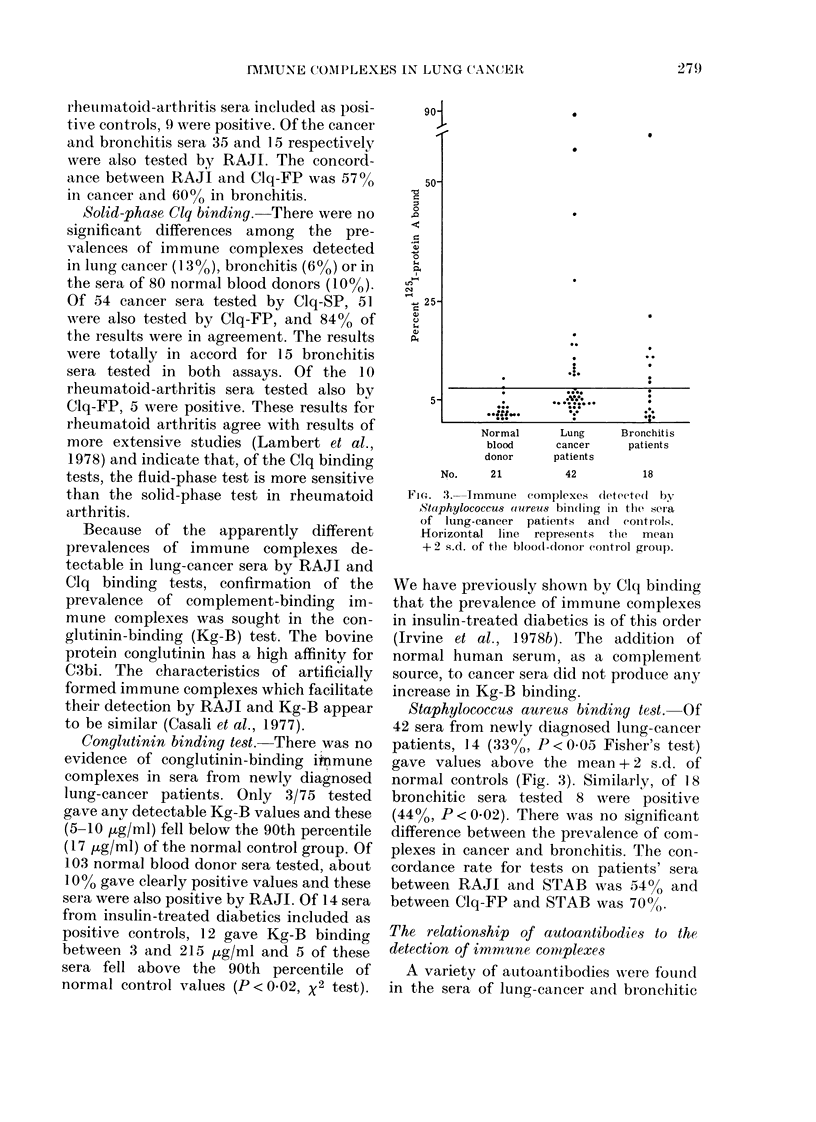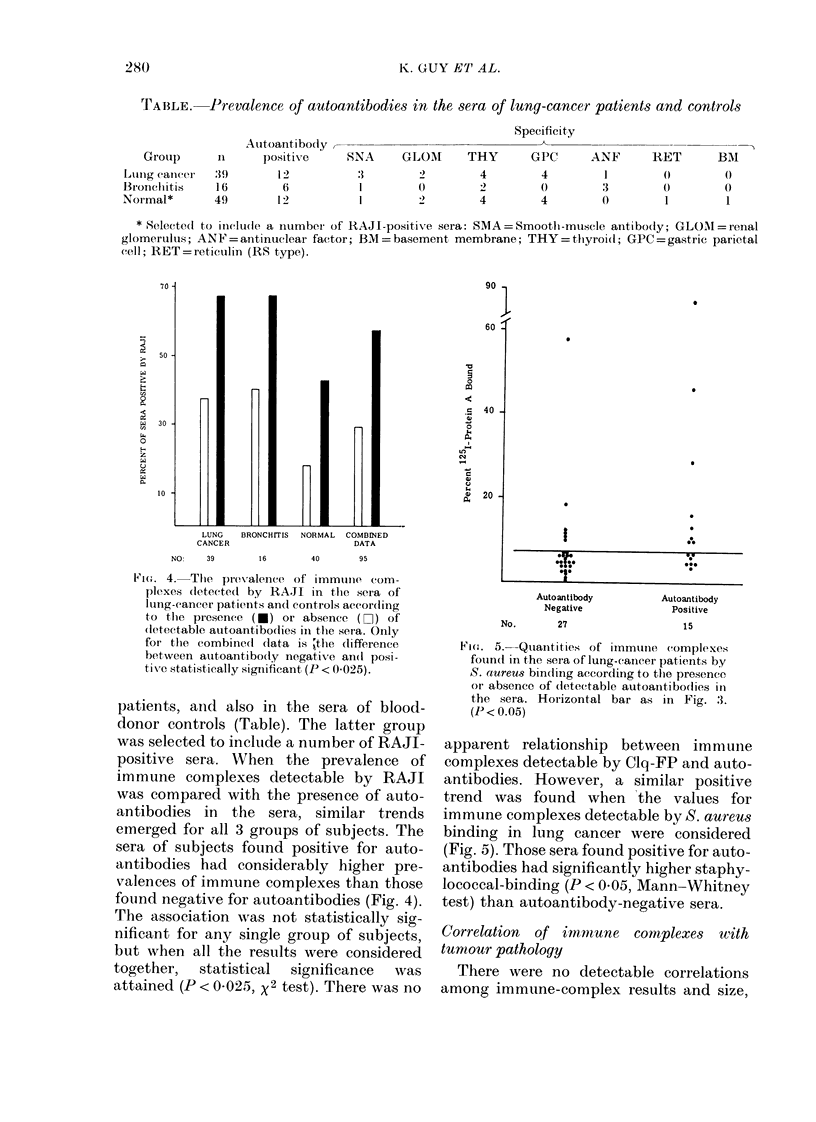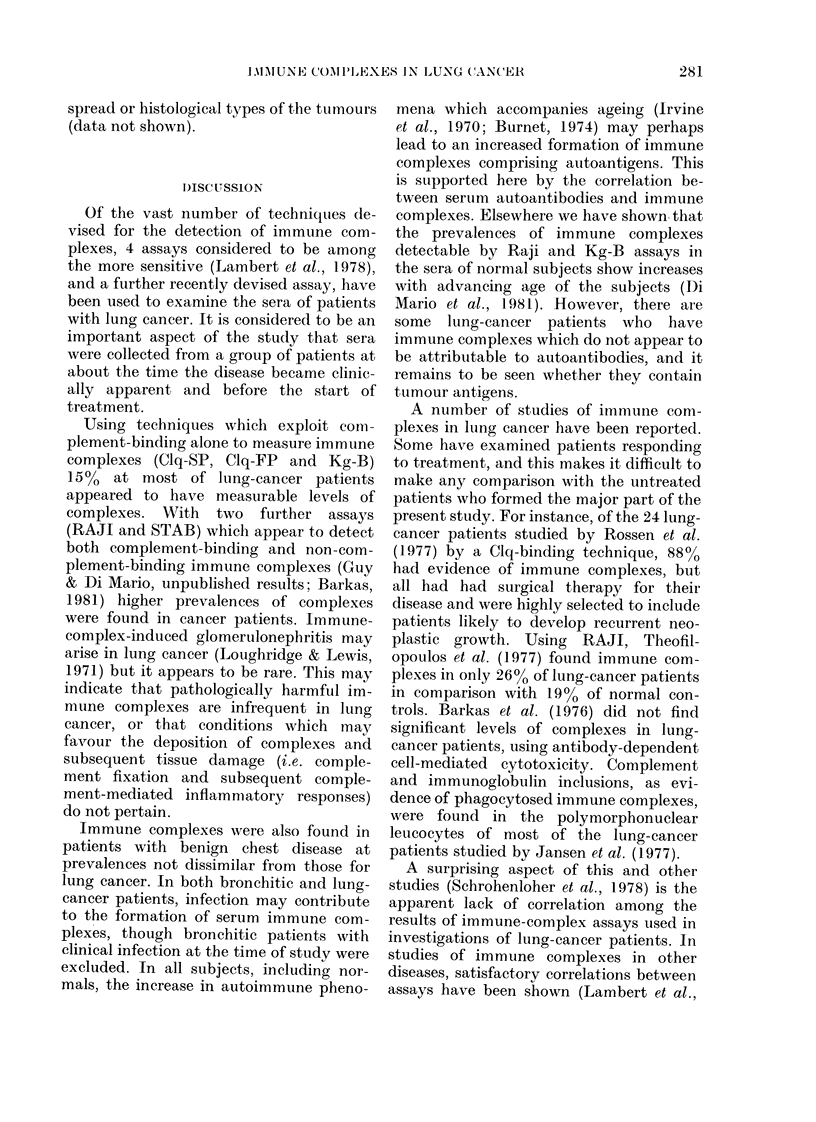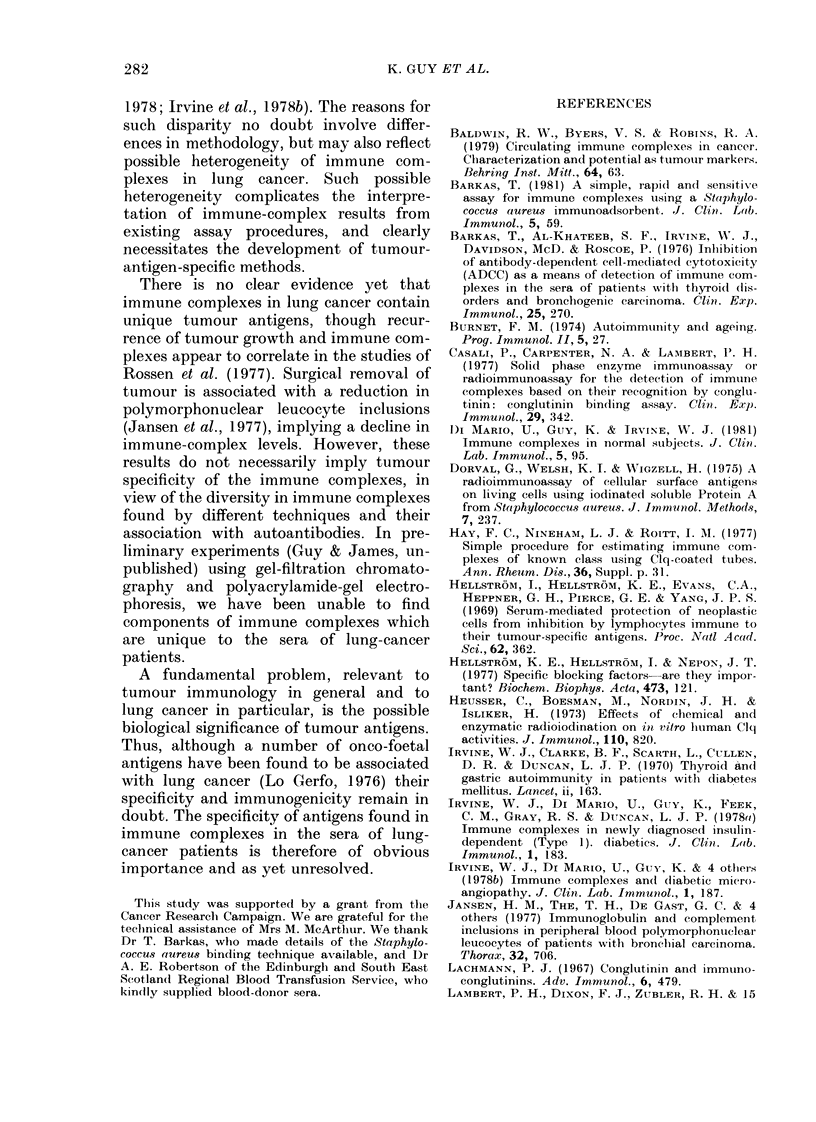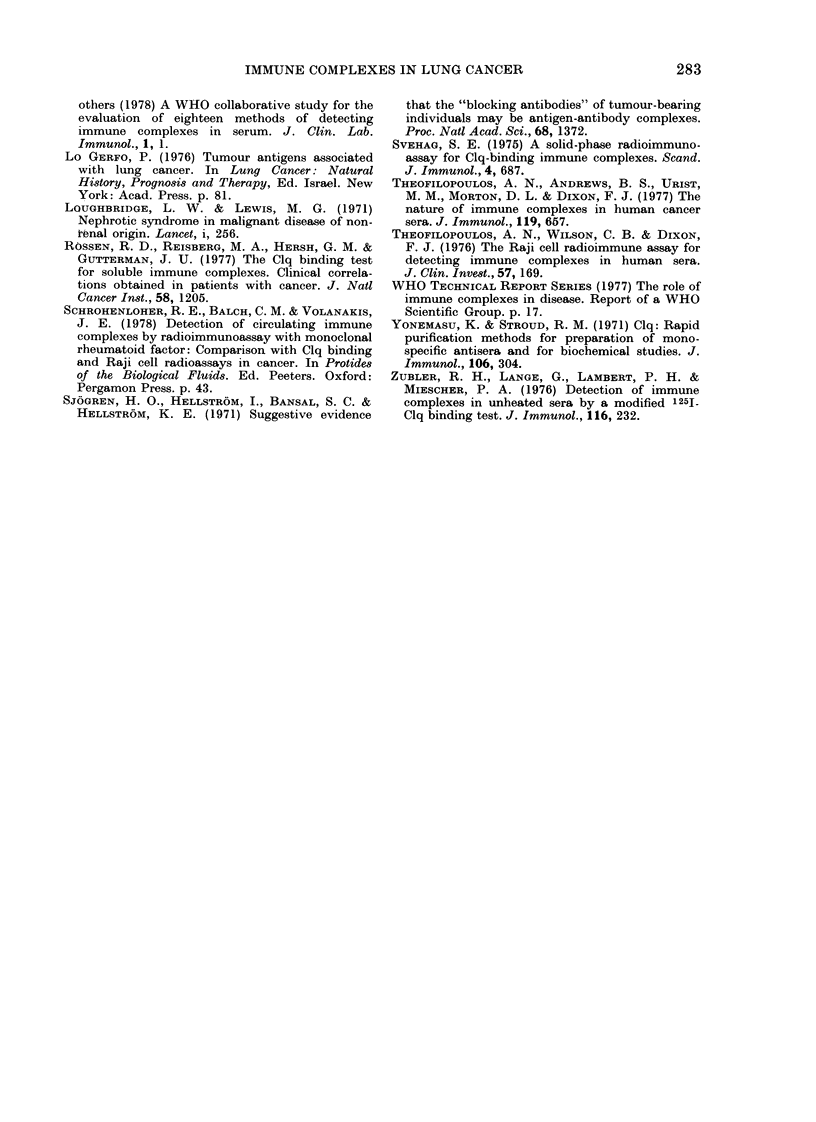# Circulating immune complexes and autoantibodies in lung cancer.

**DOI:** 10.1038/bjc.1981.45

**Published:** 1981-03

**Authors:** K. Guy, U. Di Mario, W. J. Irvine, A. M. Hunter, A. Hadley, N. W. Horne

## Abstract

The sera of 80 newly diagnosed lung-cancer patients have been examined for immune complexes and autoantibodies. Control subjects consisted of 20 bronchitic patients and 150 normal blood donors. Immune-complex measurements used 4 established and sensitive techniques (Raji cell assay, fluid and solid-phase C1q assays and conglutinin-binding assay) and a 5th newly devised technique based on the binding of polyethylene-glycol-precipitated immune-complex-rich serum fractions to Staphylococcus aureus. Using the Raji cell assay and the S. aureus binding assay to measure immune complexes, both newly diagnosed lung cancer patients and bronchitic patients had significantly higher prevalences of immune complexes than normal controls, but the two groups of patients did not differ significantly in either prevalence or quantity of immune complexes. When techniques which depend solely upon complement fixation (C1q assays and conglutinin binding) were used, only meagre quantities of immune complexes were found, and in at most 15% of newly diagnosed lung-cancer patients. The presence of autoantibodies in newly diagnosed cancer patients and controls appeared to correlate with the increase in the detectable prevalence of immune complexes.


*Br. J. Cancer* (1981) **43**, 276

### CIRCULATING IMMUNE COMPLEXES AND AUTOANTIBODIES IN LUNG CANCER

K. GUY*†, U. DI MARIO†, W. J. IRVINE†, A. M. HUNTER‡, A. HADLEY‡ AND N. W. HORNE‡

*From the †Endocrine Unit and Immunology Laboratories (Medicine), Royal Infirmary, and University Department of Medicine; and ‡Chest Unit, City Hospital, Edinburgh*

Received 30 May 1980 Accepted 10 November 1980

**Summary.—The sera of 80 newly diagnosed lung-cancer patients have been examined for immune complexes and autoantibodies. Control subjects consisted of 20 bronchitic patients and 150 normal blood donors. Immune-complex measurements used 4 established and sensitive techniques (Raji cell assay, fluid and solid-phase Clq assays and conglutinin-binding assay) and a 5th newly devised technique based on the binding of polyethylene-glycol-precipitated immune-complex-rich serum fractions to *Staphylococcus aureus*. Using the Raji cell assay and the *S. aureus* binding assay to measure immune complexes, both newly diagnosed lung cancer patients and bronchitic patients had significantly higher prevalences of immune complexes than normal controls, but the two groups of patients did not differ significantly in either prevalence or quantity of immune complexes. When techniques which depend solely upon complement fixation (Clq assays and conglutinin binding) were used, only meagre quantities of immune complexes were found, and in at most 15% of newly diagnosed lung-cancer patients. The presence of autoantibodies in newly diagnosed cancer patients and controls appeared to correlate with the increase in the detectable prevalence of immune complexes.**

THE PRESENCE of circulating immune complexes in a variety of human disorders, notably the idiopathic inflammatory diseases such as systemic lupus erythematosus and rheumatoid arthritis, is now well established. In some human malignant diseases, agents of the kind originally described as "blocking factors" by the Hellströms more than a decade ago (Hellström *et al.*, 1969) have since been recognized as complexes of antigens and antibodies (Sjögren *et al.*, 1971; Hellström *et al.*, 1977). With the advent of better techniques for their detection, apparently successful efforts have been made to find immune complexes in various human malignancies (Theofilopoulos *et al.*, 1977; Lambert *et al.*, 1978; Baldwin *et al.*, 1979). However, since the existing assays for immune complexes are antigenically non-specific, it may be expected that not all the immune complexes so far detected in malignant disease will be found to contain tumour-related antigens.

In this paper are described studies of immune complexes in the sera of a large group of patients with lung cancer, examined near to the time of clinical presentation of the disease and before the start of treatment. Of the 18 methods subjected to a recent international study (Lambert *et al.*, 1978) 4 of the more sensitive (the Raji cell radioimmunoassay, the fluid phase and solid phase Clq assays and the conglutinin-binding test) have been used to detect immune complexes. In addition, a newly devised technique based on the binding of immune complexes to *Staphylococcus aureus* has been used.

The significance attached to immune

* Present address: MRC Clinical and Population Cytogenetics Unit Western General Hospital, Crewe Road, Edinburgh.

complexes in cancer relates to the concepts, expressed in earlier studies (Hellström *et al.*, 1969; Sjögren *et al.*, 1971) that immune complexes may have unfavourable influences upon the immunity of tumour-bearing hosts. The implication of these concepts is that such complexes may comprise anti-tumour antibodies and tumour-cell antigens.

### MATERIALS AND METHODS

### *Subjects*

Sera were obtained from 80 lung-cancer patients at the City Hospital, Edinburgh. All were studied before the start of treatment and only those patients with a subsequently confirmed diagnosis of bronchogenic carcinoma were included. Normal control sera were obtained from 150 randomly selected blood donors. Sera were also obtained from 20 bronchitic patients to serve as benign chest-disease controls of comparable age to the cancer patients, and from patients with either rheumatoid arthritis or insulin-dependent diabetes to serve as positive controls for immune-complex assays. Sera were collected only from bronchitic patients clinically judged not to be suffering from infection at that time. Sera were stored in aliquots at −40°C. The age range of the lung-cancer patients was 47–84 years, of the bronchitic patients 50–82 years, and of the normal blood donors 18–58 years.

### *Immunological reagents*

Protein A (Pharmacia) was radiolabelled to a sp. act. of 20 $\mu$Ci/$\mu$g by an adaptation of the chloramine-T method (Dorval *et al.*, 1975). Human Clq was purified by the method of Yonemasu & Stroud (1971) and radiolabelled to a sp. act. of 1 $\mu$Ci/$\mu$g, using lactoperoxidase (Heusser *et al.*, 1973). Bovine conglutinin was purified by absorption to yeast (Lachmann, 1967) and further purified by DEAE-A50 chromatography. Standard preparations of aggregated Ig were tested whenever immune-complex assays were performed. Cohn Fraction II Ig was ultracentrifuged at 100,000 *g* and aggregated by heating at 63°C for 20 min. Standard preparations were made by diluting aggregated Ig from 1 mg/ml in serial doubling dilutions in normal human serum to 1–2 $\mu$g/ml.

### *Immune-complex assays*

*Raji cell radioimmunoassay* (*RAJI*).—Cells of the lymphoblastoid line Raji were cultured in RPMI-1640 containing 10% foetal calf serum, 10% tryptose-phosphate broth, antibiotic antimycotic solution and 3-(N-morpholino) propane-sulphonic acid. Viability of the harvested cells was routinely 90–95%. The assay was performed according to the method of Theofilopoulos *et al.* (1976), except that $^{125}$I-protein A (12·5 ng; 2·5 × $10^5$ ct/min) was used instead of anti-IgG to detect Raji cell-bound immune complexes. Results were expressed as $\mu$g of aggregated Ig equivalents per ml of undiluted serum, by reference to the uptake of standard preparations of aggregated Ig in normal human serum. The minimum amount of aggregated Ig equivalents readily and routinely detectable corresponded to 20 $\mu$g/ml, so 21 $\mu$g/ml was taken as the lower limit of positivity. This modification of RAJI has been used successfully in other studies (Irvine *et al.*, 1978*a*).

*Solid-phase Clq assay* (*Clq–SP*).—The technique of Svehag (1975) adapted by Hay *et al.* (1977) was used with $^{125}$I-protein A (2·5 ng; 5 × $10^4$ ct/min). Results were expressed as percentage Protein A bound, and those results exceeding the 90th percentile of normal blood-donor control values were considered positive.

*Fluid-phase Clq assay* (*Clq–FP*). The test described by Zubler *et al.* (1976) with EDTA treatment of sera was used without modification. Those percentage Clq-binding values found to exceed the mean + 2 s.d. of the mean of normal controls were considered positive.

*Conglutinin-binding test* (*Kg–B*).—The assay described by Casali *et al.* (1977) was used with $^{125}$I-protein A (5 ng; $10^5$ ct/min). Results were expressed as $\mu$g/ml of aggregated Ig equivalents, by reference to standard preparations of aggregated Ig. Those results which exceeded the 90th percentile of normal control subjects were considered positive.

*Staphylococcus aureus binding test* (*STAB*).—This is a newly devised assay, details of which are to be reported elsewhere (Barkas, 1980). Briefly, 100 $\mu$l of each serum was treated with EDTA and 2 ml of 6% polyethylene glycol 6000 (PEG) in borate–saline buffer (pH 8·4) was added. After overnight incubation at 4°C, precipitates were recovered by centrifugation, washed in 5% PEG and redissolved in phosphate-buffered saline (PBS) pH 7·2. To duplicate 100$\mu$l aliquots of resuspended material was added 100 $\mu$l of a 1% suspension of heat-killed and formalin-fixed *S. aureus* suspension in PBS plus 0·5% bovine serum albumin. After incubation at 37°C for 1 h the staphylococci were washed twice and 30 ng of $^{125}$I-Protein A was added to each tube. After 30min incubation at 4°C the staphyloccoci were washed twice and the amounts of $^{125}$I-Protein A bound were determined. Results were expressed as percent Protein A bound and results above the mean +2 s.d. of normal controls were considered positive.

*Autoantibodies.*—Standard immunofluorescence techniques with anti-human IgG and sections of rat tissues were used, except for thyroid autoantibodies, when human thyroid sections were used. Conventional tanned-red-cell tests with thyroglobulin and thyroid microsomal antigens were also used. All tests for autoantibodies were performed by individuals without knowledge of the identity of the sera or of the results of immune-complex assays.

### RESULTS

### *Immune-complex measurements in newly diagnosed lung-cancer patients and controls*

Initially, sera from newly diagnosed patients were tested by RAJI and Clq techniques. Different immune-complex assays preferentially detect subspecies of immune complexes which are defined by characteristics of the complex such as Ig class and subclass, antigen valency, complex size and the ability of the complex to activate the complement system (WHO Technical Report Series, 1977). The parallel use of two or more immune complex assays may help to avoid such methodological restrictions.

*Raji cell radioimmunoassay.*—Considerable overlap among the values obtained for patients and controls was apparent in the results, but 18/41 sera from lung-cancer patients (44%, $P < 0{\cdot}05$, Fisher's test) and 50% of 16 sera from bronchitic patients ($P < 0{\cdot}05$) gave values above 20 $\mu$g/ml in comparison with 23·5% (16) of 68 normal blood donors (Fig. 1). There were no significant differences between the values for positive lung cancer and bronchitis sera (Mann–Whitney test) or between the prevalences of immune complexes in these patients (Fisher's test).

*Fluid-phase Clq binding.*—Of 80 lung-cancer sera, 12 gave Clq binding above normal control levels (15%, $P < 0{\cdot}05$ from $\chi^2$) and 1 of 18 bronchitis sera was positive (6%, not significant) (Fig. 2). Of 10

FIG. 1.—Immune complexes detected by RAJI in the sera of lung-cancer patients and controls. Horizontal line drawn at 20 $\mu$g/ml represents the limit of positivity.

FIG. 2.—Immune complexes detected by Clq-FP in the sera of lung-cancer patients and controls. Horizontal line represents the mean + 2 s.d. of the blood-donor control.

rheumatoid-arthritis sera included as positive controls, 9 were positive. Of the cancer and bronchitis sera 35 and 15 respectively were also tested by RAJI. The concordance between RAJI and Clq-FP was 57% in cancer and 60% in bronchitis.

*Solid-phase Clq binding.*—There were no significant differences among the prevalences of immune complexes detected in lung cancer (13%), bronchitis (6%) or in the sera of 80 normal blood donors (10%). Of 54 cancer sera tested by Clq-SP, 51 were also tested by Clq-FP, and 84% of the results were in agreement. The results were totally in accord for 15 bronchitis sera tested in both assays. Of the 10 rheumatoid-arthritis sera tested also by Clq-FP, 5 were positive. These results for rheumatoid arthritis agree with results of more extensive studies (Lambert *et al.*, 1978) and indicate that, of the Clq binding tests, the fluid-phase test is more sensitive than the solid-phase test in rheumatoid arthritis.

Because of the apparently different prevalences of immune complexes detectable in lung-cancer sera by RAJI and Clq binding tests, confirmation of the prevalence of complement-binding immune complexes was sought in the conglutinin-binding (Kg-B) test. The bovine protein conglutinin has a high affinity for C3bi. The characteristics of artificially formed immune complexes which facilitate their detection by RAJI and Kg-B appear to be similar (Casali *et al.*, 1977).

*Conglutinin binding test.*—There was no evidence of conglutinin-binding immune complexes in sera from newly diagnosed lung-cancer patients. Only 3/75 tested gave any detectable Kg-B values and these (5–10 μg/ml) fell below the 90th percentile (17 μg/ml) of the normal control group. Of 103 normal blood donor sera tested, about 10% gave clearly positive values and these sera were also positive by RAJI. Of 14 sera from insulin-treated diabetics included as positive controls, 12 gave Kg-B binding between 3 and 215 μg/ml and 5 of these sera fell above the 90th percentile of normal control values ($P < 0{\cdot}02$, $\chi^2$ test).

Fig. 3.—Immune complexes detected by *Staphylococcus aureus* binding in the sera of lung-cancer patients and controls. Horizontal line represents the mean +2 s.d. of the blood-donor control group.

We have previously shown by Clq binding that the prevalence of immune complexes in insulin-treated diabetics is of this order (Irvine *et al.*, 1978*b*). The addition of normal human serum, as a complement source, to cancer sera did not produce any increase in Kg-B binding.

*Staphylococcus aureus binding test.*—Of 42 sera from newly diagnosed lung-cancer patients, 14 (33%, $P < 0{\cdot}05$ Fisher's test) gave values above the mean + 2 s.d. of normal controls (Fig. 3). Similarly, of 18 bronchitic sera tested 8 were positive (44%, $P < 0{\cdot}02$). There was no significant difference between the prevalence of complexes in cancer and bronchitis. The concordance rate for tests on patients' sera between RAJI and STAB was 54% and between Clq-FP and STAB was 70%.

### *The relationship of autoantibodies to the detection of immune complexes*

A variety of autoantibodies were found in the sera of lung-cancer and bronchitic

TABLE.—*Prevalence of autoantibodies in the sera of lung-cancer patients and controls*

| Group | n | Autoantibody positive | Specificity SNA | GLOM | THY | GPC | ANF | RET | BM |
|---|---|---|---|---|---|---|---|---|---|
| Lung cancer | 39 | 12 | 3 | 2 | 4 | 4 | 1 | 0 | 0 |
| Bronchitis | 16 | 6 | 1 | 0 | 2 | 0 | 3 | 0 | 0 |
| Normal* | 49 | 12 | 1 | 2 | 4 | 4 | 0 | 1 | 1 |

* Selected to include a number of RAJI-positive sera: SMA = Smooth-muscle antibody; GLOM = renal glomerulus; ANF = antinuclear factor; BM = basement membrane; THY = thyroid; GPC = gastric parietal cell; RET = reticulin (RS type).

FIG. 4.—The prevalence of immune complexes detected by RAJI in the sera of lung-cancer patients and controls according to the presence (■) or absence (□) of detectable autoantibodies in the sera. Only for the combined data is the difference between autoantibody negative and positive statistically significant ($P < 0{\cdot}025$).

FIG. 5.—Quantities of immune complexes found in the sera of lung-cancer patients by *S. aureus* binding according to the presence or absence of detectable autoantibodies in the sera. Horizontal bar as in Fig. 3. ($P < 0.05$)

patients, and also in the sera of blood-donor controls (Table). The latter group was selected to include a number of RAJI-positive sera. When the prevalence of immune complexes detectable by RAJI was compared with the presence of auto-antibodies in the sera, similar trends emerged for all 3 groups of subjects. The sera of subjects found positive for auto-antibodies had considerably higher prevalences of immune complexes than those found negative for autoantibodies (Fig. 4). The association was not statistically significant for any single group of subjects, but when all the results were considered together, statistical significance was attained ($P < 0{\cdot}025$, $\chi^2$ test). There was no apparent relationship between immune complexes detectable by Clq-FP and auto-antibodies. However, a similar positive trend was found when the values for immune complexes detectable by *S. aureus* binding in lung cancer were considered (Fig. 5). Those sera found positive for auto-antibodies had significantly higher staphylococcal-binding ($P < 0{\cdot}05$, Mann–Whitney test) than autoantibody-negative sera.

### *Correlation of immune complexes with tumour pathology*

There were no detectable correlations among immune-complex results and size, spread or histological types of the tumours (data not shown).

### DISCUSSION

Of the vast number of techniques devised for the detection of immune complexes, 4 assays considered to be among the more sensitive (Lambert *et al.*, 1978), and a further recently devised assay, have been used to examine the sera of patients with lung cancer. It is considered to be an important aspect of the study that sera were collected from a group of patients at about the time the disease became clinically apparent and before the start of treatment.

Using techniques which exploit complement-binding alone to measure immune complexes (Clq-SP, Clq-FP and Kg-B) 15% at most of lung-cancer patients appeared to have measurable levels of complexes. With two further assays (RAJI and STAB) which appear to detect both complement-binding and non-complement-binding immune complexes (Guy & Di Mario, unpublished results; Barkas, 1981) higher prevalences of complexes were found in cancer patients. Immune-complex-induced glomerulonephritis may arise in lung cancer (Loughridge & Lewis, 1971) but it appears to be rare. This may indicate that pathologically harmful immune complexes are infrequent in lung cancer, or that conditions which may favour the deposition of complexes and subsequent tissue damage (*i.e.* complement fixation and subsequent complement-mediated inflammatory responses) do not pertain.

Immune complexes were also found in patients with benign chest disease at prevalences not dissimilar from those for lung cancer. In both bronchitic and lung-cancer patients, infection may contribute to the formation of serum immune complexes, though bronchitic patients with clinical infection at the time of study were excluded. In all subjects, including normals, the increase in autoimmune phenomena which accompanies ageing (Irvine *et al.*, 1970; Burnet, 1974) may perhaps lead to an increased formation of immune complexes comprising autoantigens. This is supported here by the correlation between serum autoantibodies and immune complexes. Elsewhere we have shown that the prevalences of immune complexes detectable by Raji and Kg-B assays in the sera of normal subjects show increases with advancing age of the subjects (Di Mario *et al.*, 1981). However, there are some lung-cancer patients who have immune complexes which do not appear to be attributable to autoantibodies, and it remains to be seen whether they contain tumour antigens.

A number of studies of immune complexes in lung cancer have been reported. Some have examined patients responding to treatment, and this makes it difficult to make any comparison with the untreated patients who formed the major part of the present study. For instance, of the 24 lung-cancer patients studied by Rossen *et al.* (1977) by a Clq-binding technique, 88% had evidence of immune complexes, but all had had surgical therapy for their disease and were highly selected to include patients likely to develop recurrent neoplastic growth. Using RAJI, Theofilopoulos *et al.* (1977) found immune complexes in only 26% of lung-cancer patients in comparison with 19% of normal controls. Barkas *et al.* (1976) did not find significant levels of complexes in lung-cancer patients, using antibody-dependent cell-mediated cytotoxicity. Complement and immunoglobulin inclusions, as evidence of phagocytosed immune complexes, were found in the polymorphonuclear leucocytes of most of the lung-cancer patients studied by Jansen *et al.* (1977).

A surprising aspect of this and other studies (Schrohenloher *et al.*, 1978) is the apparent lack of correlation among the results of immune-complex assays used in investigations of lung-cancer patients. In studies of immune complexes in other diseases, satisfactory correlations between assays have been shown (Lambert *et al.*,

1978; Irvine *et al.*, 1978*b*). The reasons for such disparity no doubt involve differences in methodology, but may also reflect possible heterogeneity of immune complexes in lung cancer. Such possible heterogeneity complicates the interpretation of immune-complex results from existing assay procedures, and clearly necessitates the development of tumour-antigen-specific methods.

There is no clear evidence yet that immune complexes in lung cancer contain unique tumour antigens, though recurrence of tumour growth and immune complexes appear to correlate in the studies of Rossen *et al.* (1977). Surgical removal of tumour is associated with a reduction in polymorphonuclear leucocyte inclusions (Jansen *et al.*, 1977), implying a decline in immune-complex levels. However, these results do not necessarily imply tumour specificity of the immune complexes, in view of the diversity in immune complexes found by different techniques and their association with autoantibodies. In preliminary experiments (Guy & James, unpublished) using gel-filtration chromatography and polyacrylamide-gel electrophoresis, we have been unable to find components of immune complexes which are unique to the sera of lung-cancer patients.

A fundamental problem, relevant to tumour immunology in general and to lung cancer in particular, is the possible biological significance of tumour antigens. Thus, although a number of onco-foetal antigens have been found to be associated with lung cancer (Lo Gerfo, 1976) their specificity and immunogenicity remain in doubt. The specificity of antigens found in immune complexes in the sera of lung-cancer patients is therefore of obvious importance and as yet unresolved.

This study was supported by a grant from the Cancer Research Campaign. We are grateful for the technical assistance of Mrs M. McArthur. We thank Dr T. Barkas, who made details of the *Staphylococcus aureus* binding technique available, and Dr A. E. Robertson of the Edinburgh and South East Scotland Regional Blood Transfusion Service, who kindly supplied blood-donor sera.

### REFERENCES

BALDWIN, R. W., BYERS, V. S. & ROBINS, R. A. (1979) Circulating immune complexes in cancer. Characterization and potential as tumour markers. *Behring Inst. Mitt.*, **64**, 63.

BARKAS, T. (1981) A simple, rapid and sensitive assay for immune complexes using a *Staphylococcus aureus* immunoadsorbent. *J. Clin. Lab. Immunol.*, **5**, 59.

BARKAS, T., AL-KHATEEB, S. F., IRVINE, W. J., DAVIDSON, McD. & ROSCOE, P. (1976) Inhibition of antibody-dependent cell-mediated cytotoxicity (ADCC) as a means of detection of immune complexes in the sera of patients with thyroid disorders and bronchogenic carcinoma. *Clin. Exp. Immunol.*, **25**, 270.

BURNET, F. M. (1974) Autoimmunity and ageing. *Prog. Immunol. II*, **5**, 27.

CASALI, P., CARPENTER, N. A. & LAMBERT, P. H. (1977) Solid phase enzyme immunoassay or radioimmunoassay for the detection of immune complexes based on their recognition by conglutinin: conglutinin binding assay. *Clin. Exp. Immunol.*, **29**, 342.

DI MARIO, U., GUY, K. & IRVINE, W. J. (1981) Immune complexes in normal subjects. *J. Clin. Lab. Immunol.*, **5**, 95.

DORVAL, G., WELSH, K. I. & WIGZELL, H. (1975) A radioimmunoassay of cellular surface antigens on living cells using iodinated soluble Protein A from *Staphylococcus aureus*. *J. Immunol. Methods*, **7**, 237.

HAY, F. C., NINEHAM, L. J. & ROITT, I. M. (1977) Simple procedure for estimating immune complexes of known class using Clq-coated tubes. *Ann. Rheum. Dis.*, **36**, Suppl. p. 31.

HELLSTRÖM, I., HELLSTRÖM, K. E., EVANS, C.A., HEPPNER, G. H., PIERCE, G. E. & YANG, J. P. S. (1969) Serum-mediated protection of neoplastic cells from inhibition by lymphocytes immune to their tumour-specific antigens. *Proc. Natl Acad. Sci.*, **62**, 362.

HELLSTRÖM, K. E., HELLSTRÖM, I. & NEPOM, J. T. (1977) Specific blocking factors—are they important? *Biochem. Biophys. Acta*, **473**, 121.

HEUSSER, C., BOESMAN, M., NORDIN, J. H. & ISLIKER, H. (1973) Effects of chemical and enzymatic radioiodination on *in vitro* human Clq activities. *J. Immunol.*, **110**, 820.

IRVINE, W. J., CLARKE, B. F., SCARTH, L., CULLEN, D. R. & DUNCAN, L. J. P. (1970) Thyroid and gastric autoimmunity in patients with diabetes mellitus. *Lancet*, ii, 163.

IRVINE, W. J., DI MARIO, U., GUY, K., FEEK, C. M., GRAY, R. S. & DUNCAN, L. J. P. (1978*a*) Immune complexes in newly diagnosed insulin-dependent (Type 1). diabetics. *J. Clin. Lab. Immunol.*, **1**, 183.

IRVINE, W. J., DI MARIO, U., GUY, K. & 4 others (1978*b*) Immune complexes and diabetic microangiopathy. *J. Clin. Lab. Immunol.*, **1**, 187.

JANSEN, H. M., THE, T. H., DE GAST, G. C. & 4 others (1977) Immunoglobulin and complement inclusions in peripheral blood polymorphonuclear leucocytes of patients with bronchial carcinoma. *Thorax*, **32**, 706.

LACHMANN, P. J. (1967) Conglutinin and immunoconglutinins. *Adv. Immunol.*, **6**, 479.

LAMBERT, P. H., DIXON, F. J., ZUBLER, R. H. & 15

others (1978) A WHO collaborative study for the evaluation of eighteen methods of detecting immune complexes in serum. *J. Clin. Lab. Immunol.*, **1**, 1.

LO GERFO, P. (1976) Tumour antigens associated with lung cancer. In *Lung Cancer: Natural History, Prognosis and Therapy*, Ed. Israel. New York: Acad. Press. p. 81.

LOUGHBRIDGE, L. W. & LEWIS, M. G. (1971) Nephrotic syndrome in malignant disease of non-renal origin. *Lancet*, i, 256.

RÓSSEN, R. D., REISBERG, M. A., HERSH, G. M. & GUTTERMAN, J. U. (1977) The Clq binding test for soluble immune complexes. Clinical correlations obtained in patients with cancer. *J. Natl Cancer Inst.*, **58**, 1205.

SCHROHENLOHER, R. E., BALCH, C. M. & VOLANAKIS, J. E. (1978) Detection of circulating immune complexes by radioimmunoassay with monoclonal rheumatoid factor: Comparison with Clq binding and Raji cell radioassays in cancer. In *Protides of the Biological Fluids*. Ed. Peeters. Oxford: Pergamon Press. p. 43.

SJÖGREN, H. O., HELLSTRÖM, I., BANSAL, S. C. & HELLSTRÖM, K. E. (1971) Suggestive evidence that the "blocking antibodies" of tumour-bearing individuals may be antigen-antibody complexes. *Proc. Natl Acad. Sci.*, **68**, 1372.

SVEHAG, S. E. (1975) A solid-phase radioimmunoassay for Clq-binding immune complexes. *Scand. J. Immunol.*, **4**, 687.

THEOFILOPOULOS, A. N., ANDREWS, B. S., URIST, M. M., MORTON, D. L. & DIXON, F. J. (1977) The nature of immune complexes in human cancer sera. *J. Immunol.*, **119**, 657.

THEOFILOPOULOS, A. N., WILSON, C. B. & DIXON, F. J. (1976) The Raji cell radioimmune assay for detecting immune complexes in human sera. *J. Clin. Invest.*, **57**, 169.

WHO TECHNICAL REPORT SERIES (1977) The role of immune complexes in disease. Report of a WHO Scientific Group. p. 17.

YONEMASU, K. & STROUD, R. M. (1971) Clq: Rapid purification methods for preparation of monospecific antisera and for biochemical studies. *J. Immunol.*, **106**, 304.

ZUBLER, R. H., LANGE, G., LAMBERT, P. H. & MIESCHER, P. A. (1976) Detection of immune complexes in unheated sera by a modified $^{125}$I-Clq binding test. *J. Immunol.*, **116**, 232.